# Are there any sociodemographic factors associated with non-uptake of HPV vaccination of girls in high-income countries with school-based vaccination programmes? A systematic review

**DOI:** 10.1136/jech-2024-222488

**Published:** 2024-12-22

**Authors:** Emily Dema, Roeann Osman, Kate Soldan, Nigel Field, Pam Sonnenberg

**Affiliations:** 1 University College London Institute for Global Health, London, UK; 2 UK Health Security Agency, London, UK

**Keywords:** VACCINATION, INFECTIONS, Health inequalities, SYSTEMATIC REVIEW

## Abstract

**Background:**

Uptake of human papillomavirus (HPV) vaccination is generally high in high-income countries with school-based vaccination programmes; however, lower uptake in certain population subgroups could continue pre-immunisation inequalities in cervical cancer.

**Methods:**

Six electronic databases were searched for quantitative articles published between 1 September 2006 and 20 February 2023, which were representative of the general population, with individual-level data on routine school-based vaccination (with >50% coverage) and sociodemographic measures. Titles, abstracts and full-text articles were screened for eligibility criteria and assessed for bias. A second independent reviewer randomly screened 20% of articles at each stage. A narrative synthesis summarised findings.

**Results:**

24 studies based in eight countries (Australia, Belgium, Canada, New Zealand, Norway, Sweden, Switzerland, UK) were included. Studies reported vaccination uptake by individual-level and area-level socioeconomic status (SES), parental education, religion, ethnicity and/or country of birth. 19 studies reported that more than 70% were vaccinated (range: 50.7%–93.0%). Minority ethnic groups and migrants were more likely to have lower vaccination uptake than White groups and non-migrants (11/11 studies). Lower SES was also associated with lower uptake of vaccination (11/17 studies). Associations with other sociodemographic characteristics, such as parental education and religion, were less clear.

**Conclusions:**

Even in high-income countries with high coverage school-based vaccination programmes, inequalities are seen. The totality of available evidence suggests girls from lower SES and minority ethnic groups tend to be less likely to be vaccinated. Findings could inform targeted approaches to mop-up vaccination and cervical cancer screening amidst changing HPV epidemiology in a vaccine era.

**Trial registration number:**

CRD42023399648.

**WHAT IS ALREADY KNOWN ON THIS TOPIC**

- Many high-income countries are on track to achieve the WHO Cervical Cancer Elimination goals, but inequalities in cervical cancer prevention remain. Previous reviews have demonstrated that certain sociodemographic factors are associated with lower uptake of human papillomavirus (HPV) vaccination, including ethnicity and healthcare coverage. However, no study has focused on high-income settings with high vaccination coverage and school-based delivery, which should be the most equitable and effective programme structure.

**WHAT THIS STUDY ADDS**

- This study uses a robust systematic review methodology that identified 24 population-representative studies of factors (socioeconomic status, education, ethnicity and religion) associated with HPV vaccination in girls in eight high-income countries with school-based vaccination programmes. Across these studies, a narrative synthesis identified lower uptake of vaccination was consistently associated with lower socioeconomic status and among ethnic minority groups and migrants, though associations were minor or inconsistent for other factors. This work demonstrates that even in high-income countries with high vaccination coverage and school-based vaccination, inequalities may need to be addressed.

**HOW THIS STUDY MIGHT AFFECT RESEARCH, PRACTICE OR POLICY**

- High vaccination coverage and changing HPV epidemiology will require changes to cervical cancer screening programmes, and it is important that unvaccinated groups do not get left behind. These findings can inform studies of the outcomes of vaccination, and targeting of mop-up vaccination and cervical screening to help achieve WHO Cervical Cancer Elimination targets.

## Introduction

 Human papillomavirus (HPV) vaccination uptake is generally high in high-income countries with school-based vaccination programmes, with many such countries on track to reach the WHO Cervical Cancer Elimination target of 90% coverage; however, even small differences in access might be important.^1^ Globally, HPV vaccination of girls has been introduced in 75 of 84 high-income countries (as of 2021), 41 of which have fully or partially school-based vaccination programmes (online supplemental figure 1).^2 3^ Many countries have also now introduced gender-neutral vaccination. Prevaccination, cervical cancer has been associated with lower socioeconomic status (SES) in some high-income countries, with the combined effects of higher risks of HPV infection and disease, as well as lower attendance for cervical screening in some population subgroups.^4 5^

Previous research has demonstrated that school-based programmes are more equitable than other forms of vaccination programmes, such as clinic-based programmes,^6^ but inequalities can still occur. Lower uptake of HPV vaccinations in certain population subgroups could continue the inequalities in cervical cancer prevention that were observed prevaccination.^7^ Groups with lower HPV vaccination rates may also be less likely to attend cervical cancer screening, as well as be at higher risk for high-risk HPV infection,^8^ thereby exacerbating risk of cervical cancer within a population largely protected due to the success of vaccination and screening programmes.^7^

A previous systematic review of studies primarily in the USA demonstrated evidence of differences in HPV vaccination by ethnicity and healthcare coverage but found no association with parental education or family income.^9^ However, HPV vaccination in the USA is offered in healthcare clinics and paid for using health insurance, meaning this review cannot be extrapolated to countries with free-of-charge school-based vaccination programmes, such as the UK. Additionally, this review was conducted in 2013, and therefore captured only early experience of HPV vaccination programmes.

This review and narrative synthesis, conducted in 2023, therefore aimed to determine whether any characteristics were associated with not being vaccinated for HPV among girls in high-income countries with high coverage and school-based vaccination programmes, which we would expect to be the most effective and equitable. Population-based studies which reported on individual-level HPV vaccination status among girls by at least one sociodemographic measure were included. Findings could inform current and future school-based vaccination programmes globally to aid in achieving the WHO Cervical Cancer Elimination target.

## Methods

### Conduct and protocol

This review was conducted in accordance with the Preferred Reporting Items for Systematic Reviews and Meta-Analyses (PRISMA) guidelines, and the protocol was published on the International Prospective Register of Systematic Reviews (PROSPERO) in advance (CRD42023399648).

### Search strategy

Six electronic databases (MEDLINE, Web of Science, Scopus, Embase, PsycINFO and The Cochrane Library) were searched for articles published between 1 September 2006 (initial licensure of first HPV vaccine) and 20 February 2023. Searches were conducted separately for each database using database-specific searches. An initial search was conducted to identify relevant keywords and indexed terms in titles and abstracts. Search strings were developed based on three key concepts: HPV vaccination, sociodemographic factors (eg, ethnicity, parental education, religion, socioeconomic factors) and high-income countries with school-based vaccination programmes. Countries were identified based on those listed as ‘high-income’ according to the World Bank.^10^ Based on this list, vaccination programme structure was ascertained using global summary reports, as well as national health agency websites.^11 12^ Medical Subject Headings (MeSH) were used where relevant, along with truncated free text and subject headings. The search terms used are shown in online supplemental table 1. Searches were supplemented by searching of reference lists.

### Screening

All identified references were exported to Zotero and deduplicated. The first reviewer (ED) conducted title and abstract screening for all results, as well as full text screening for articles included at that stage. A second independent reviewer (RO) screened 20% of articles in each stage, selected randomly. Conflicts were resolved and consensus reached through discussions between the two reviewers at each stage. There was very minimal disagreement between reviewers at each stage.

### Inclusion and exclusion criteria

Study inclusion and exclusion criteria were defined using a PICOS (population, intervention, comparator, outcome and study design) table, described in table 1.

**Table 1:** Systematic review inclusion and exclusion criteria (PICOS table)

	Inclusion criteria	Exclusion criteria
Population	Vaccine-eligible girls living in high income countries with school-based (or partially school-based) vaccination programmes (Andorra, Australia, Austria, Bahamas, Barbados, Belgium, Brunei, Canada, Channel Islands, Chile, Croatia, Curacao, Cyprus, Estonia, Finland, Gibraltar, Guam, Hong Kong, Hungary, Iceland, Ireland, Isle of Man, Israel, Latvia, Liechtenstein, Macau, New Zealand, Northern Mariana Islands, Norway, Panama, Puerto Rico, Saint Kitts and Nevis, Seychelles, Singapore, Slovenia, Spain, Sweden, Switzerland, Taiwan, Trinidad and Tobago and the UK (Britain, England, Wales, Scotland and Northern Ireland))	Studies focusing on LMICs, vaccination programmes which are not school-based, no individual-level data, vaccine coverage lower than 50%*
Intervention	Barriers and facilitators to vaccine uptake; determinants of not being vaccinatedStudies which report on HPV vaccination by **at least one** sociodemographic measure (parental education, area-level deprivation, income/socioeconomic status, ethnicity, country of birth, religion)	Studies which do not report HPV vaccination by sociodemographic characteristics; studies reporting intent to vaccinate
Comparator	N/A	N/A
Outcome	HPV vaccination (at least one dose), individual-level data	Studies reporting only on catch-up vaccination
Study design	Representative of population, primary research, quantitative findings, conference abstractsPublished 1 September 2006 to 20 February 2023	Reviews, commentaries, editorials, case reports, guidelines, data not representative of the general population (based on sampling methodology)
Other	Published in English	

* Vaccination coverage was included as a criterion because the factors associated with non-uptake in a low coverage setting will not be the same as those in higher coverage settings. However, most settings with school-based vaccination are expected to have high coverage.

HPV, human papillomavirus; LMICs, low and middle income countries; PICOS, population, intervention, comparator, outcome and study design.

### Quality appraisal

Included full-text studies were appraised using relevant STROBE (Strengthening the reporting of observational studies in epidemiology) checklists. The first reviewer assessed the quality of all papers using the Joanna Briggs Institute (JBI) critical appraisal checklist^13^ for prevalence studies (20% by second reviewer). A meeting was held to discuss any disagreements. Each article was assigned yes, no or unclear for a set of nine criteria (including representativeness, recruitment, sample size, descriptions of participants, response rate, measurement bias, consistency and appropriateness), then given an overall appraisal (include, exclude or seek further information) (online supplemental figure 2). To distinguish between studies using data from vaccination records and those which used self or parent-reported vaccination status, studies were classified as ‘yes’ for ‘Identification of vaccination’ only where a study used data from vaccination records, and ‘unclear’ for those with self-report. Previous research suggests high agreement between parental report and vaccination records for HPV vaccination.^14^

### Data extraction

Data for all included articles were extracted by the first reviewer, and 20% by the second reviewer as a consistency check. Following the bias assessment, a narrative synthesis was used to describe included studies according to country and characteristics associated with being unvaccinated. Findings were summarised according to the following sociodemographic characteristics: area-level deprivation, income, education, religion, and ethnicity/country of birth.

## Results

### Screening

**Figure 1:**
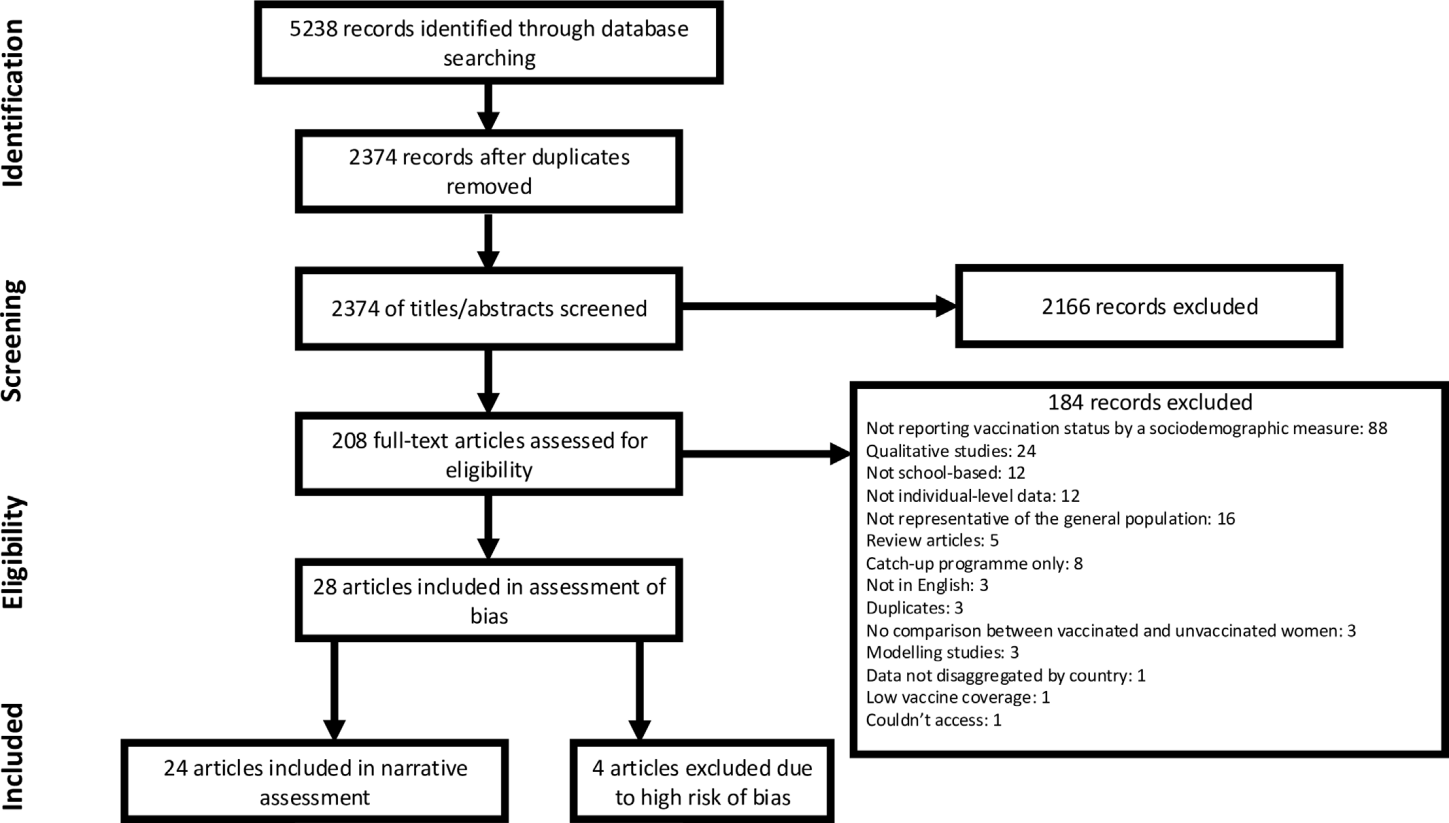
PRISMA diagram showing the screening and selection process for systematic review. HPV, human papillomavirus; PRISMA, Preferred Reporting Items for Systematic Reviews and Meta-Analyses.

Of 5238 records initially identified through the database search, 2374 titles and abstracts were reviewed following deduplication, and 208 full-text articles assessed for eligibility (figure 1).

Full-text studies were excluded for a variety of reasons (shown in figure 1), but most commonly due to not reporting vaccination status by a sociodemographic measure (n=88).

### Quality appraisal

The JBI critical appraisal checklist^13^ for prevalence studies was used to assess 28 articles (online supplemental table 2). Four articles were excluded. Two of the excluded studies were available only as conference abstracts, so sufficient information was not available to determine study quality. Another study was excluded because of missing data in the analysis (70% of respondents were not included), which introduced potential selection bias. The final excluded study was identified as being unrepresentative of the general population. Excluded studies had at least three categories rated as ‘unclear’.

### Study characteristics

Of 24 included studies, the majority were conducted in the UK (n=6), Canada (n=6) and Australia (n=4), followed by Norway (n=3), Sweden (n=2), New Zealand (n=1), Belgium (n=1) and Switzerland (n=1) (table 2). Most studies were conducted during the first decade following introduction of HPV vaccination, with the earliest data collection period in 2007.^615^,^20^ The most recent data collection period was 2020.^21^

**Table 2:** Characteristics of included studies

Study	Country	Year of data collection	Time since HPV vaccination programme started (years)	Population	Study type	Sample size	At least one dose of HPV vaccine (%)
Brotherton *et al*^15^	Australia	2007–2011	0–4	Vaccine-eligible women (aged <26 in 2007) attending cervical cancer screening in Victoria	Registry/records	289 477	54.0
Gertig *et al*^16^	Australia	2007–2011	0–4	Vaccine-eligible women (aged <17 in 2007) attending cervical cancer screening in Victoria	Registry/records	38 956	63.8
Mak *et al*^22^	Australia	2009–2010	2–3	Girls in Year 7 in Western Australia	Registry/records	21 181	74.3
Brotherton *et al*^21^	Australia	2020	13	Girls born in 2005 in the Australian Immunisation Register	Registry/records	137 916	87.1
Lefevere *et al*^17^	Belgium	2007–2012	0–3	Girls born in 1998 and 1999 who have National Alliance of Christian Mutualities health insurance	Registry/records	66 664	90.0
Remes *et al*^19^	Canada	2007–2011	0–4	Vaccine eligible girls (Grade 8) in Ontario	Registry/records	144 047	50.7
Smith *et al*^18^	Canada	2007–2009	0–2	Vaccine eligible girls (Grade 8) in Ontario	Registry/records	2519	56.5
Ogilvie *et al*^35^	Canada	2008–2009	1–2	Girls enrolled in Grade 6 (age 11) in British Columbia	Cross sectional survey	2025	65.1
Gilbert *et al*^23^	Canada	2013–2014	6–7	Girls aged 12–14 years old	Cross sectional survey	5720	72.3
Carpiano *et al*^24^	Canada	2016–2017	9–10	Girls aged 12–14 years old	Cross sectional survey	5213	70.9
Krawczyk *et al*^25^	Canada	2010	3	Girls aged 9–10 years in Quebec	Cross sectional survey	774	88.2
Poole *et al*^26^	New Zealand	2009	1	Vaccine-eligible girls from schools in the Auckland District Health Board catchment area	Registry/records	8665	71.5
Bjerke *et al*^27^	Norway	2009–2014	0–5	Girls born between 1997 and 2002 who were registered in the Norwegian Central Population Registry	Registry/records	177 387	72.5–87.3
Feiring *et al*^28^	Norway	2009–2012	0–3	Girls born between 1997 and 1999 who were registered in the Norwegian Central Population Registry	Registry/records	84 139	78.3
Hansen *et al*^29^	Norway	2009–2011	0–2	Girls born between 1997 and 1999 who were eligible for routine vaccination	Registry/records	90 842	78.2
Wang *et al*^6^	Sweden	2007–2014	0–2	Girls born in Sweden between 1990 and 2003 who were living in Sweden between 2007 and 2014	Registry/records	207 467	79.0
Wemrell *et al*^30^	Sweden	2013–2020	1–8	Girls aged 2–7 years old in 2010, followed up at 10–12 years old	Registry/records	311 656	81.2
Riesen *et al*^37^	Switzerland	2009–2016	1–8	Girls aged 14–17 years in the Swiss National Vaccination Coverage Survey	Cross-sectional survey	8965	53.2
Fisher *et al*^36^	UK	2008–2011	0–3	Girls born between 1995 and 1998 from three primary care trusts in South West England	Registry/records	14 282	88.6
Sinka *et al*^31^	UK	2008–2011	0–3	Girls eligible for HPV vaccination in Scotland from 2008 to 2011	Registry/records	86 769	91.0–93.0
Roberts *et al*^20^	UK	2007–2008	0	Girls in Year 8 attending two UK primary care trusts in Greater Manchester	Registry/records	2817	~70*
Spencer *et al*^32^	UK	2008–2009	0–1	Girls eligible for vaccination in 2008–2009 in the North West of England	Registry/records	56 234	82.4
Bedford *et al*^33^	UK	2015–2016	7–8	Girls from the Millennium Cohort Study who were aged 14 years old in 2012 (born 2000–2002)	Cross-sectional survey	5695	92.3
Bowyer *et al*^34^	UK	2012	4	Girls aged 15–16 years old (year 11) in 13 London schools	Cross-sectional survey	1912	78.0†

* Article reports only disaggregated uptake.

† Vaccination completion rather than initiation.

HPV, human papillomavirus.

Uptake of at least one dose of the HPV vaccination was consistently high across studies, with most reporting greater than 70% vaccinated.^69 17 20^,^34^ Of the studies reporting lower vaccination rates (50%–70%),^15 16 18 19 35^ most were conducted in the initial years of HPV vaccination programmes in Canada and Australia.

Of the included studies, 17 used vaccination and sociodemographic data from national registries or vaccination and census records,^615^,^22 26^ while seven used data from cross-sectional surveys.^23^,^2533^ Sample sizes were large for all studies, with 14 studies reporting a sample size larger than 10 000.^615^,^17 19 21 22 27^ The smallest sample size was 774 girls,^25^ and the largest was 311 656 girls.^30^

**Figure 2:**
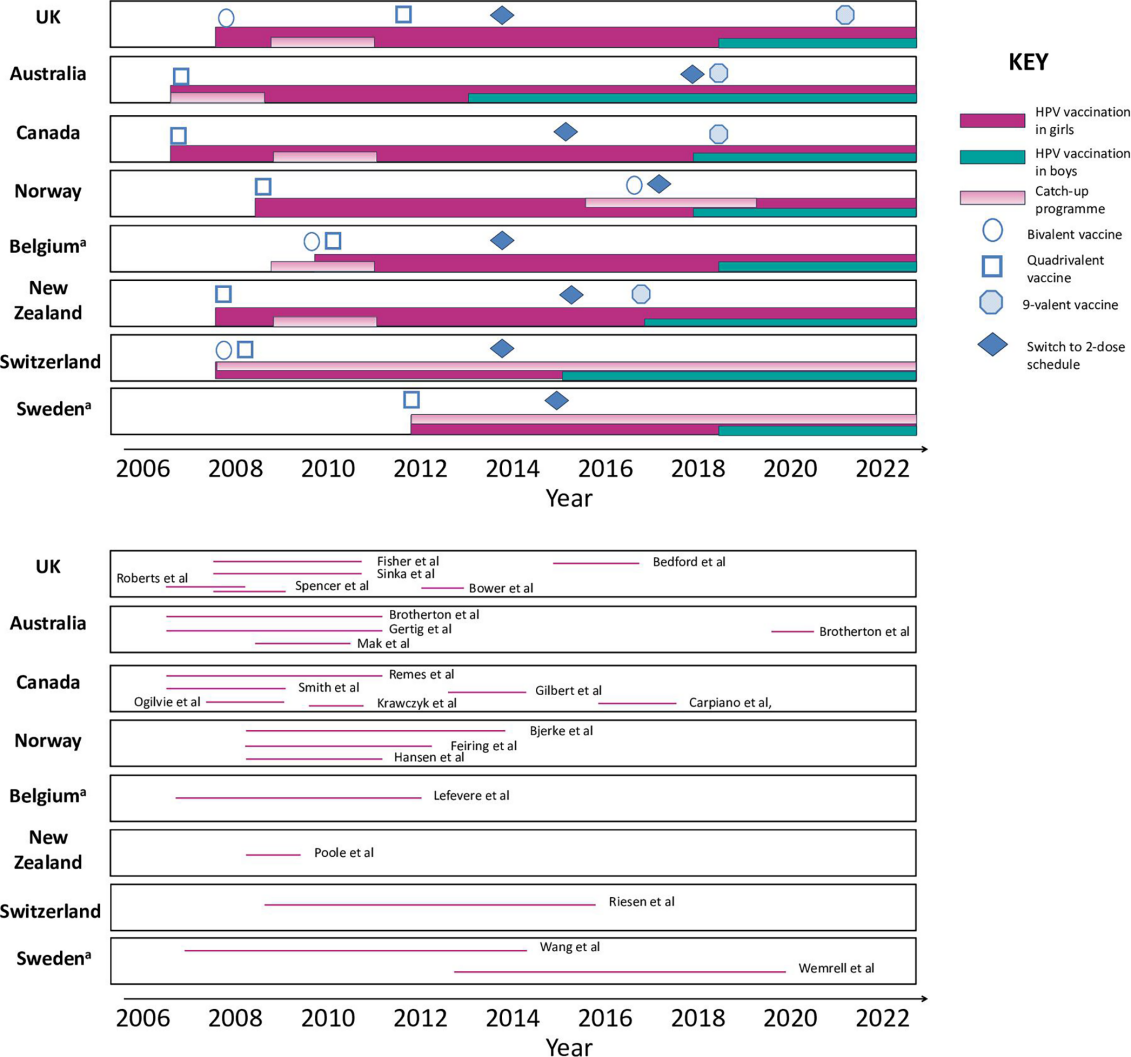
Timeline of (**A**) HPV vaccination programmes in eight included countries (UK, Canada, Australia, Norway, New Zealand, Belgium, Switzerland and Sweden) and (**B**) data collection periods for 24 included studies. ^a^HPV vaccination offered earlier than demonstrated in the diagram. The diagram illustrates when the free vaccinations became available and offered in schools. HPV, human papillomavirus

HPV vaccination programme structures are similar across the eight countries represented in the included papers (figure 2). In most of the eight countries, at the time of the studies, routine vaccination had been offered to girls aged 11–13 years, while catch-up vaccination had been offered to older girls in their later teens to early 20s. In all these countries/studies, HPV vaccination was free-of-charge at time of school-based vaccination.

### Factors associated with non-uptake of vaccination

#### Socio-economic status

All studies, except one,^35^ examined the association between vaccination and SES (table 3): 17 of these 23 reported an association.^615^,^20 22 26^ Thirteen studies used individual-level indicators of family or parental income (online supplemental table 3),^617 18 23^,^25 27^ while 11 studies used non-individual-level indicators of SES, including area-level deprivation^15 16 20 21 31 32 36 37^ and school-level SES (online supplemental table 4).^22 26^ One study examined both income and area-level deprivation.^19^ Generally, lower SES (as indicated by family income, area-level deprivation or school-level SES) was associated with lower uptake of HPV vaccination. However, six studies (UK,^32 36^ Switzerland,^37^ New Zealand^26^ and Canada^18 19^) found higher vaccination uptake among those with lower SES, though the strengths of these associations were not consistent. Strengths of association between SES and vaccination uptake were similar between studies with individual-level versus area-level measures.

**Table 3:** Summary of risk factors for non-uptake of HPV vaccination in included studies

Study	Country	Individual-level SES	Area or school-level SES	Education	Religion	Ethnicity	Country of birth
Brotherton *et al* (2015)^15^	Australia						
Mak *et al*^22^	Australia		↑↑				
Brotherton *et al* (2022)^21^	Australia		↑↑				
Gertig *et al*^16^	Australia		↑↑				
Lefevere *et al*^17^	Belgium	↑↑					
Krawczyk *et al*^25^	Canada				Higher vax: Christian vs non-Christian	Higher vax: White vs non-White	
Smith *et al*^18^	Canada	↑↓					
Remes *et al*^19^	Canada	↑↓	↑↓				
Ogilvie *et al*^35^	Canada			↑↓			
Gilbert *et al*^23^	Canada						Lower vax: born abroad
Carpiano *et al*^24^	Canada						
Poole *et al*^26^	New Zealand		↑↓			Higher vax: Pacific girls	
Hansen *et al*^29^	Norway	↑↑		↑↓			
Feiring *et al*^28^	Norway	↑↑		↑↓			
Bjerke *et al*^27^	Norway	↑↑		↑↓			Lower vax: born abroad
Wang *et al*^6^	Sweden	↑↑		↑↑			Lower vax: born abroad
Wemrell *et al*^30^	Sweden	↑↑		↑↑			Lower vax: born abroad
Riesen *et al*^37^	Switzerland		↑↓				
Bowyer *et al*^34^	UK				Higher vax: non-religious vs Christian	Lower vax: Black/other vs White	
Roberts *et al*^20^	UK		↑↑			Lower vax: other ethnic groups vs White	
Spencer *et al*^32^	UK		↑↓			Lower vax: Black/Asian/other vs White	
Fisher *et al*^36^	UK		↑↓			Lower vax: Black/Asian/Chinese vs White	
Sinka *et al*^31^	UK						
Bedford *et al*^33^	UK	↑↑			Lower vax: any religion vs non-religious	Lower vax: Black/other vs White	

Key: 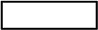, variable not analysed; 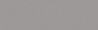, no association found; 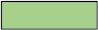, association present; 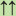, association present: higher SES or education, higher vaccination; 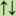, association present: lower SES or education, higher vaccination.

HPV, human papillomavirus; SES, socioeconomic status; Vax, vaccination.

#### Parental education

Nine studies examined the association between parental education and vaccination uptake (table 3). Four were in Canada,^23 24 35^ three in Norway^27^,^29^ and two in Sweden.^6 30^ Of these, six reported an association between parental education and HPV vaccination uptake, though the direction of this association differed (online supplemental table 5). Among the studies in Norway, as well as one in Canada,^35^ higher parental education was associated with reduced HPV vaccination uptake. Both Swedish studies reported that lower HPV vaccination uptake was associated with lower parental education.

#### Religion

Six studies analysed whether there was an association between religion and HPV vaccination; four studies used individual-level religious identity,^2533^,^35^ while two studies reported school-level religious affiliation (table 3).^22 31^ Associations were mixed. Two studies reported no association between religion and vaccination.^31 35^ Some studies found higher vaccination uptake among more religious groups compared with non-religious groups. An Australian study found higher uptake in Catholic schools compared with secular government schools. Similarly, a Canadian study found increased vaccination uptake among Christian girls compared with non-Christian girls. Two UK studies reported reduced vaccination among religious groups,^33 34^ however, these associations did not remain following adjusted analysis. Most associations between religion and vaccination were weak (online supplemental table 6).

#### Ethnicity or country of birth

Eleven of 12 studies which examined ethnicity or country of birth identified an association between these variables and HPV vaccination (table 3).^620 23 25^,^27 30 32^ Of these, two studies from Canada, two from Sweden and one from Norway reported on country of birth, rather than ethnicity. Strength of association between ethnicity or country of birth, and HPV vaccination for each study is summarised in online supplemental table 7. The association with vaccination was not significant in one of the Canadian studies (p>0.05),^35^ but the other Canadian study^23^ found girls whose parents were born outside of Canada were more likely to be unvaccinated against HPV. The Swedish studies also found non-vaccination was higher among girls born outside of Sweden, or whose parents were born outside of Sweden, compared with Swedish-born girls.^6 30^ The Norwegian study found that girls with at least one parent born in Norway were most likely to receive HPV vaccination.^27^

All seven studies which reported on ethnicity identified an association with HPV vaccination: one study was from Canada,^25^ one from New Zealand^26^ and the remainder from the UK.^2032^,^34 36^ In the Canadian study, uptake was higher among White girls compared with non-White girls, and in the New Zealand study, Pacific girls had higher vaccination rates than Asian, Maori and European girls. Among the UK studies, all reported that ethnic minority groups, especially Black/Black British and other ethnic groups, were less likely to be vaccinated than White groups. Two of these studies adjusted for SES.^33 36^ Generally, there was a strong trend for reduced vaccination among minority ethnic groups and immigrants in all studies where an association was identified.

## Discussion

This systematic review and narrative synthesis of 24 papers from high-income countries with high coverage school-based HPV vaccination programmes highlights some sociodemographic factors that have been associated with lower uptake of the vaccination, including area or school-level SES (area-level deprivation), individual-level SES (income) and ethnicity, and some with variable and weaker association, including parental education and religion. Importantly, this review demonstrates that, even in high-income countries with high vaccination coverage and school-based delivery, which should be more equitable than other programme designs, potentially important inequalities in vaccination uptake can be seen: these should be considered carefully in vaccination impact studies.

Lower uptake of HPV vaccination was associated with a lower SES across most (11/17) of the studies that identified an association between these variables. Additionally, where studies examined ethnicity and country of birth, minority ethnic groups and migrants were found (11/12) to have lower vaccination coverage than White groups and non-migrants. Associations with other sociodemographic characteristics, such as parental education and religion, were less clear. Some characteristics, such as education, may reflect ‘U-shaped’ associations, where lower education levels and higher education levels may both be associated with non-uptake of vaccination in different settings.

Some of our findings are similar to a previous systematic review,^9^ which found ethnicity to be associated with HPV vaccination initiation, though no associations with parental education or family income were observed. However, our systematic review builds on the previous review by focusing analysis on countries with high coverage school-based vaccination programmes, and limits potential bias attributable to different healthcare systems and lower vaccination coverage that feature in US studies. All included studies had large, representative samples with the findings generalisable to eligible girls in the respective countries. Additionally, many of the studies used registry data and vaccination records, minimising reporting or selection bias given our restriction to studies with limited missing data and a high proportion of data linkage. There may still be some misclassification and bias in the studies we included; however, by restricting inclusion as we did we believe we minimised this making our findings as robust as possible.

There are also some potential weaknesses to consider. First, it is possible that relevant articles were not captured in the search strategy, though this is mitigated by the finding that no further studies were identified by reviewing reference lists of included studies and other relevant systematic reviews. Second, the included studies are from eight countries, but there are at least 33 other high-income countries with school-based vaccination programmes (online supplemental figure 1). Therefore, while this study aimed to produce generalisable findings, not all countries had data available, so a certain amount of caution is needed in applying trends to other countries. Additionally, many of these studies were conducted within the first 5 years of each country’s HPV vaccination programme, so updated analyses would be warranted to determine changes in inequalities over time, as well as to include analysis of gender-neutral vaccination. This review does not include data on vaccination in boys, despite many of the included countries now implementing gender-neutral vaccination. However, this review chose to focus on girls to robustly look at inequalities in vaccination over time, as there is not yet enough comparable data on vaccination in boys, though trends will likely be quite similar, so these findings can still inform gender-neutral vaccination programmes. Additionally, the study uses ‘at least one dose’ of HPV vaccination as the outcome, so findings can inform single-dose vaccination programmes, even though some of the included studies were conducted at a time where countries had a 2-dose or 3-dose regimen, and the effect of one-dose schedules on uptake equitability will certainly need to be monitored. This review focuses only on sociodemographic characteristics associated with HPV vaccination, rather than other childhood vaccinations, because HPV vaccination is unique from other childhood vaccinations due to the older age at vaccination, as well as the association with sexual activity. However, evidence suggests uptake of other school-based vaccinations (Td/IPV and MenACWY) offered to adolescents in England, as an example of a high-income country, is similar to, if not slightly lower than, uptake of HPV vaccination.^38^ Many countries, including the UK, also framed HPV vaccination as an ‘anticancer’ vaccine rather than an ‘anti-STI’ vaccine to avoid the association with attitudes toward sexual behaviour.

Access to raw, individual-level data to conduct a meta-analysis may also strengthen this work. However, a meta-analysis was not conducted due to the wide variation in study design across the included studies and the lack of individual-level data, which might bias the associations. However, in many studies, vaccination coverage was not presented by population group as a proportion, instead showing only associations with non-uptake (eg, ORs). Rather, study-level associations have been presented and compared. Displaying findings by country, as we have, allows readers to make their own judgements regarding which countries’ evidence is most relevant to them.

### Implications for policy

Despite high coverage of HPV vaccination achieved in countries with routine, school-based vaccination programmes, this systematic review demonstrates that inequalities can still occur, with lower SES and minority ethnic groups generally less likely to be vaccinated. Evidence of inequalities in access to vaccination must be considered in the context of herd protection and infection risk. If groups not accessing HPV vaccination are not within the same sexual networks as vaccinated individuals and therefore not protected by indirect effects, or at higher risk for HPV infection due to sexual behaviours, then they may face an even larger increased risk for developing cervical cancer. The combined effects of lower uptake, exposure risk and indirect protection are what matter ultimately: studies of the impact of vaccination should therefore consider these population groups for subanalyses. However, meanwhile, the lower uptake should prompt consideration of mitigating measures.

The changing HPV epidemiology due to vaccination will require major changes to cervical cancer screening programmes in the coming years. Intervals between screens will likely be lengthened for vaccinated cohorts; however, unvaccinated women may still require more frequent cervical screening, and/or a more targeted approach to improve participation in cervical screening. Vaccination and screening programmes may wish to consider implementing campaigns specific to migrants and ethnic minority groups to increase awareness of vaccination in schools and screening opportunities. Additionally, health authorities might consider offering additional vaccination and screening opportunities in areas of lower SES. The use of self-sampling for cervical cancer screening may also help to increase uptake among undervaccinated and underscreened groups.^39^ A recent systematic review found that culturally sensitivity interventions, information campaigns and self-sampling were promising methods to achieve higher screening uptake among migrant women.^40^

Therefore, this review’s identification of key groups at risk for lower vaccination uptake as minority ethnic groups and those with lower SES, could inform studies of vaccination outcomes, targeting of mop-up vaccination and targeting of screening to improve disease prevention among these unvaccinated groups and achieve the WHO Cervical Cancer Elimination goals.

## Supplementary material

10.1136/jech-2024-222488 online supplemental file 1

## Data Availability

Data sharing not applicable as no datasets generated and/or analysed for this study.

## References

[1] eClinicalMedicine (2023). Global strategy to eliminate cervical cancer as a public health problem: are we on track?. EClinMed.

[2] Our World in Data (2022). Vaccination schedule for human papillomavirus vaccine. https://ourworldindata.org/grapher/human-papillomavirus-vaccine-immunization-schedule?tab=table.

[3] PATH (2022). Global HPV vaccine introduction overview. https://media.path.org/documents/Global_Vaccine_Intro_Overview_Slides_Final_PATHwebsite_MAR_2022_qT92Wwh.pdf.

[4] Shack L, Jordan C, Thomson CS (2008). Variation in incidence of breast, lung and cervical cancer and malignant melanoma of skin by socioeconomic group in England. BMC Cancer.

[5] Broberg G, Wang J, Östberg A-L (2018). Socio-economic and demographic determinants affecting participation in the Swedish cervical screening program: A population-based case-control study. PLoS One.

[6] Wang J, Ploner A, Sparén P (2019). Mode of HPV vaccination delivery and equity in vaccine uptake: A nationwide cohort study. Prev Med.

[7] Johnson HC, Lafferty EI, Eggo RM (2018). Effect of HPV vaccination and cervical cancer screening in England by ethnicity: a modelling study. Lancet Public Health.

[8] Tanton C, Soldan K, Beddows S (2015). High-Risk Human Papillomavirus (HPV) Infection and Cervical Cancer Prevention in Britain: Evidence of Differential Uptake of Interventions from a Probability Survey. Cancer Epidemiol Biomarkers Prev.

[9] Fisher H, Trotter CL, Audrey S (2013). Inequalities in the uptake of human papillomavirus vaccination: a systematic review and meta-analysis. Int J Epidemiol.

[10] World Bank (2023). High income. https://data.worldbank.org/country/XD.

[11] De Oliveira LH, Janusz CB, Da Costa MT (2022). HPV vaccine introduction in the Americas: a decade of progress and lessons learned. Expert Rev Vaccines.

[12] Colzani E, Johansen K, Johnson H (2021). Human papillomavirus vaccination in the European Union/European Economic Area and globally: a moral dilemma. Euro Surveill.

[13] Munn Z, Moola S, Lisy K, Aromataris E, Munn Z (2020). JBI Manual for Evidence Synthesis.

[14] Walton S, Cortina-Borja M, Dezateux C (2017). Measuring the timeliness of childhood vaccinations: Using cohort data and routine health records to evaluate quality of immunisation services. Vaccine (Auckl).

[15] Brotherton JML, Malloy M, Budd AC (2015). Effectiveness of less than three doses of quadrivalent human papillomavirus vaccine against cervical intraepithelial neoplasia when administered using a standard dose spacing schedule: Observational cohort of young women in Australia. Papillomavirus Res.

[16] Gertig DM, Brotherton JML, Budd AC (2013). Impact of a population-based HPV vaccination program on cervical abnormalities: a data linkage study. BMC Med.

[17] Lefevere E, Theeten H, Hens N (2015). From non school-based, co-payment to school-based, free Human Papillomavirus vaccination in Flanders (Belgium): A retrospective cohort study describing vaccination coverage, age-specific coverage and socio-economic inequalities. Vaccine (Auckl).

[18] Smith LM, Brassard P, Kwong JC (2011). Factors associated with initiation and completion of the quadrivalent human papillomavirus vaccine series in an Ontario cohort of grade 8 girls. BMC Public Health.

[19] Remes O, Smith LM, Alvarado-Llano BE (2014). Individual- and regional-level determinants of human papillomavirus (HPV) vaccine refusal: the Ontario Grade 8 HPV vaccine cohort study. BMC Public Health.

[20] Roberts SA, Brabin L, Stretch R (2011). Human papillomavirus vaccination and social inequality: results from a prospective cohort study. Epidemiol Infect.

[21] Brotherton J, Hendry A, Dey A (2022). HPV vaccination coverage: slightly improved two-dose schedule completion estimates and historical estimates lower on AIR than HPV Register. Aust N Z J Public Health.

[22] Mak DB, Bulsara MK, Wrate MJ (2013). Factors determining vaccine uptake in Western Australian adolescents. J Paediatr Child Health.

[23] Gilbert NL, Gilmour H, Dubé È (2016). Estimates and determinants of HPV non-vaccination and vaccine refusal in girls 12 to 14 y of age in Canada: Results from the Childhood National Immunization Coverage Survey, 2013. Hum Vaccin Immunother.

[24] Carpiano RM, Polonijo AN, Gilbert N (2019). Socioeconomic status differences in parental immunization attitudes and child immunization in Canada: Findings from the 2013 Childhood National Immunization Coverage Survey (CNICS). Prev Med.

[25] Krawczyk A, Knäuper B, Gilca V (2015). Parents’ decision-making about the human papillomavirus vaccine for their daughters: I. Quantitative results. Hum Vaccin Immunother.

[26] Poole T, Goodyear-Smith F, Petousis-Harris H (2012). Human papillomavirus vaccination in Auckland: Reducing ethnic and socioeconomic inequities. Vaccine (Auckl).

[27] Bjerke RD, Laake I, Feiring B (2021). Time trends in HPV vaccination according to country background: a nationwide register-based study among girls in Norway. BMC Public Health.

[28] Feiring B, Laake I, Molden T (2015). Do parental education and income matter? A nationwide register-based study on HPV vaccine uptake in the school-based immunisation programme in Norway. BMJ Open.

[29] Hansen BT, Campbell S, Burger E (2015). Correlates of HPV vaccine uptake in school-based routine vaccination of preadolescent girls in Norway: A register-based study of 90,000 girls and their parents. Prev Med.

[30] Wemrell M, Vicente RP, Merlo J (2023). Mapping sociodemographic and geographical differences in human papillomavirus non-vaccination among young girls in Sweden. Scand J Public Health.

[31] Sinka K, Kavanagh K, Gordon R (2014). Achieving high and equitable coverage of adolescent HPV vaccine in Scotland. J Epidemiol Community Health.

[32] Spencer AM, Roberts SA, Brabin L (2014). Sociodemographic factors predicting mother’s cervical screening and daughter’s HPV vaccination uptake. J Epidemiol Community Health.

[33] Bedford H, Firman N, Waller J (2021). Which young women are not being vaccinated against HPV? Cross-sectional analysis of a UK national cohort study. Vaccine (Auckl).

[34] Bowyer HL, Dodd RH, Marlow LAV (2014). Association between human papillomavirus vaccine status and other cervical cancer risk factors. Vaccine (Auckl).

[35] Ogilvie G, Anderson M, Marra F (2010). A population-based evaluation of a publicly funded, school-based HPV vaccine program in British Columbia, Canada: parental factors associated with HPV vaccine receipt. PLoS Med.

[36] Fisher H, Audrey S, Mytton JA (2014). Examining inequalities in the uptake of the school-based HPV vaccination programme in England: a retrospective cohort study. J Public Health (Oxf).

[37] Riesen M, Konstantinoudis G, Lang P (2018). Exploring variation in human papillomavirus vaccination uptake in Switzerland: a multilevel spatial analysis of a national vaccination coverage survey. BMJ Open.

[38] UK Health Security Agency (2022). Human papillomavirus (HPV) vaccination coverage in adolescents in England. https://www.gov.uk/government/statistics/human-papillomavirus-hpv-vaccine-coverage-estimates-in-england-2022-to-2023/human-papillomavirus-hpv-vaccination-coverage-in-adolescents-in-england-2022-to-2023.

[39] Pretsch PK, Spees LP, Brewer NT (2023). Effect of HPV self-collection kits on cervical cancer screening uptake among under-screened women from low-income US backgrounds (MBMT-3): a phase 3, open-label, randomised controlled trial. Lancet Public Health.

[40] Alam Z, Cairns JM, Scott M (2023). Interventions to increase cervical screening uptake among immigrant women: A systematic review and meta-analysis. PLoS One.

