# Supplementary material for: Are there any sociodemographic factors associated with non-uptake of HPV vaccination of girls in high-income countries with school-based vaccination programmes? A systematic review
Source: J Epidemiol Community Health. 2024 Dec 22;79(5):e222488. doi: 10.1136/jech-2024-222488 (PMC12015032; doi:10.1136/jech-2024-222488)
Supplement: online supplemental file 1 [file jech-79-5-s001.docx]

**Supplementary Material**

**Supplementary Figure 1. HPV vaccination in high-income countries^a^**


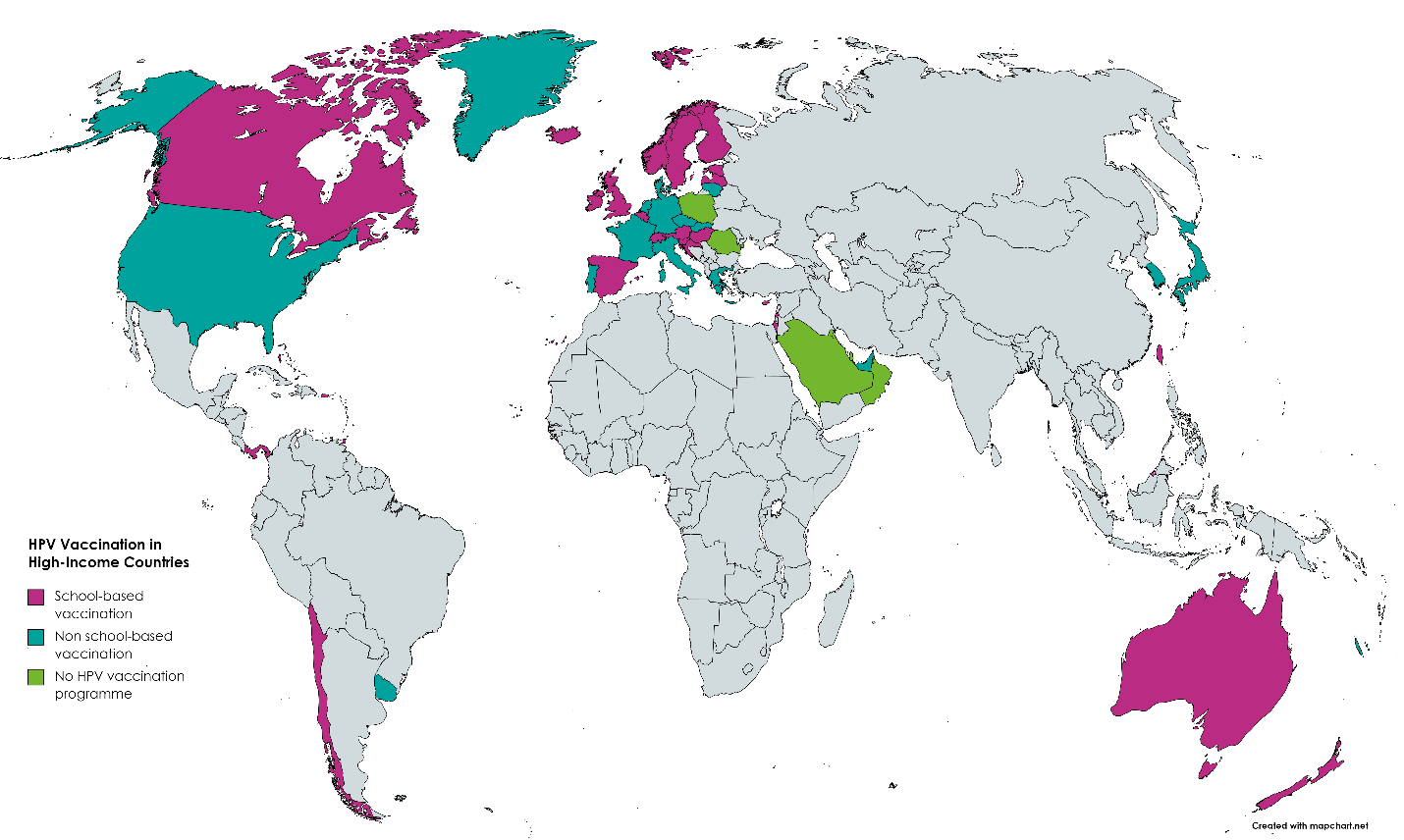


^a^ School-based programmes may also include vaccination in other settings, such as healthcare clinics. High-income countries based on World Bank classification as of 2021. School-based programmes based on literature search as of February 2023.

**Supplementary Figure 2. JBI critical appraisal checklist**^1^

**
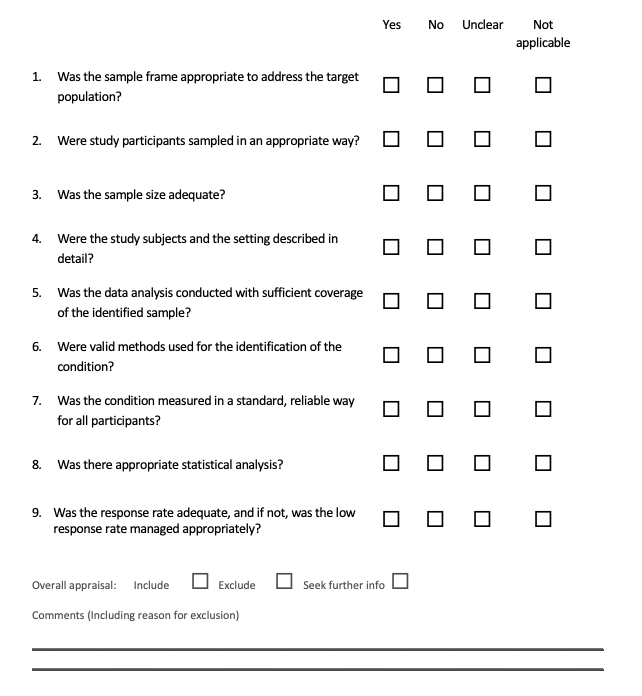
**

**Supplementary Table 1. Search terms**

| **Topic*** | **Keywords** | **MeSH terms (where applicable)** |
| --- | --- | --- |
| HPV | hpv or human papillomavirus or papillomavirus or human papilloma virus | Papillomavirus Vaccines |
| Vaccination | vaccin* or immune* | Papillomavirus Vaccines |
| Sociodemographic factors | ethnic* or religi* or income or socioeconomic or sociodemographic or disparit* or inequal* or unequal* or educat* or race | Healthcare Disparities or Ethnicity or Religion or Socioeconomic Factors or Sociodemographic Factors or Health Status Disparities or Social Class or Education |
| School-based vaccination programmes | (Andorra or Australia or Austria or Bahamas or Barbados or Belgium or Brunei or Canada or Channel Islands or Chile or Croatia or Curacao or Cyprus or Estonia or Finland or Gibraltar or Guam or Hong Kong or Hungary or Iceland or Ireland or Isle of Man or Israel or Latvia or Liechtenstein or Macau or New Zealand or Northern Mariana Islands or Norway or Panama or Puerto Rico or (Saint Kitts and Nevis) or Seychelles or Singapore or Slovenia or Spain or Sweden or Switzerland or Taiwan or (Trinidad and Tobago) or United Kingdom or UK or Britain or England or Wales or Scotland or Northern Ireland)  or  grade or school or school-based |  |

*All topics combined using “AND” within search engines

**Supplementary Table 2. Bias assessment using JBI critical appraisal checklist**

| **Major Components** | Sample frame | Sampling | Sample size | Study subject description | Sample coverage | Identification of vaccination | Reliable measurement | Statistical analysis | Response rate | **Overall appraisal** |
| --- | --- | --- | --- | --- | --- | --- | --- | --- | --- | --- |
| Bedford et al^2^ | Yes | Yes | Yes | Yes | Yes | Unclear | Yes | Yes | Yes | Include |
| Bjerke et al^3^ | Yes | Yes | Yes | Yes | Yes | Yes | Yes | Yes | Yes | Include |
| Bowyer et al^4^ | Unclear | Yes | Yes | Yes | Yes | Unclear | Yes | Yes | Yes | Include |
| Brotherton et al (2022)^5^ | Yes | Yes | Yes | Yes | Yes | Yes | Yes | Unclear | Yes | Include |
| Brotherton et al (2015)^6^ | Yes | Yes | Yes | Yes | Yes | Yes | Yes | Yes | Yes | Include |
| Carpiano et al^7^ | Yes | Unclear | Yes | Yes | Yes | Unclear | Yes | Yes | Yes | Include |
| Feiring et al^8^ | Yes | Yes | Yes | Yes | Yes | Yes | Yes | Yes | Yes | Include |
| Fisher et al^9^ | Yes | Yes | Yes | Yes | Yes | Yes | Yes | Yes | Yes | Include |
| Gertig et al^10^ | Yes | Yes | Yes | Yes | Yes | Yes | Unclear | Unclear | Yes | Include |
| Gilbert et al^11^ | Yes | Yes | Yes | Yes | Yes | Yes | Unclear | Yes | Yes | Include |
| Hansen et al^12^ | Yes | Yes | Yes | Yes | Yes | Yes | Yes | Yes | Yes | Include |
| Krawczyk et al^13^ | Yes | Yes | Yes | Yes | Yes | Unclear | Yes | Yes | Yes | Include |
| Lefevere et al^14^ | Yes | Unclear | Yes | Yes | Yes | Yes | Yes | Unclear | Yes | Include |
| Mak et al^15^ | Yes | Yes | Yes | Unclear | Yes | Yes | Yes | Yes | Yes | Include |
| Meghani et al^16^ | Yes | Unclear | Unclear | Yes | Unclear | Unclear | Yes | Unclear | Unclear | Exclude |
| Ogilvie et al^17^ | Yes | Yes | Yes | Yes | Yes | Unclear | Yes | Yes | Yes | Include |
| Pollock et al^18^ | Unclear | Unclear | Yes | Yes | Yes | Yes | Yes | Yes | Unclear | Exclude |
| Poole et al^19^ | Yes | Yes | Yes | Yes | Yes | Yes | Yes | Unclear | Yes | Include |
| Remes et al^20^ | Yes | Yes | Yes | Yes | Yes | Yes | Yes | Yes | Yes | Include |
| Riesen et al^21^ | Yes | Unclear | Yes | Yes | Yes | Yes | Unclear | Yes | Yes | Include |
| Roberts et al^22^ | Unclear | Yes | Yes | Yes | Yes | Yes | Yes | Yes | Yes | Include |
| Shapiro et al^23^ | Yes | Yes | Unclear | Yes | Yes | Unclear | Yes | Yes | Unclear | Exclude |
| Sinka et al^24^ | Yes | Yes | Yes | Yes | Yes | Yes | Yes | Unclear | Yes | Include |
| Smith et al^25^ | Yes | Yes | Yes | Yes | Yes | Yes | Yes | Yes | Yes | Include |
| Spencer et al^26^ | Yes | Unclear | Yes | Yes | Unclear | Yes | Yes | Yes | Yes | Include |
| Wang et al^27^ | Yes | Yes | Yes | Yes | Unclear | Yes | Yes | Unclear | Yes | Include |
| Wemrell et al^28^ | Yes | Yes | Yes | Yes | Yes | Yes | Yes | Yes | Yes | Include |
| Yeung et al^29^ | Yes | Yes | Yes | Unclear | Unclear | Unclear | Yes | Yes | Unclear | Exclude |

**Supplementary Table 3. Strength of association between HPV vaccination and individual-level socioeconomic status (SES) among included studies which identified an association between these variables**

| **Study** | **Country** | **Sociodemographic measure** | **Measure of association** | **Outcome** | **Adjusted for?** | **Reference group** | **Result** | **Summary** |
| --- | --- | --- | --- | --- | --- | --- | --- | --- |
| Lefevere et al^14^ | Belgium | Household income (based on Beneficiary of Increase Reimbursement) | Proportion with confidence intervals | Vaccination | N/A | Low-income girls | 0.91 (95%CI 0.90-0.91) high income v 0.81 (0.78-0.83) low income | Higher proportion initiating vaccination among higher income girls compared to lower income girls |
| Smith et al^25^ | Canada | Neighbourhood income quintile | Adjusted Odds Ratio | Vaccination | Age, income, urban/rural, vaccination history, health service utilisation | Third income quintile | 0.75 (95%CI: 0.57-0.99) | Girls in the fourth income quintile were less likely to receive the vaccine than those in the middle (third) income quintile |
| Remes et al^20^ | Canada | Neighbourhood income quintile | Adjusted Odds Ratio | Vaccine refusal | Vaccination history, healthcare utilisation, medical history, area level deprivation | 3^rd^ income quartile | Lowest income quartile, 1.13 (95%CI 1.08-1.17); highest income quartile 1.21 (1.17-1.25) | Those in the lowest income quartile and highest income quartile were more likely to refuse vaccination compared to the 3^rd^ quartile. |
| Hansen et al^12^ | Norway | Maternal income | Adjusted Odds Ratio | Vaccination | Age, marital status, occupational status, maternal country of birth, children in household, maternal cervical screening, region, year of birth | Intermediate income | Lowest income bracket, 0.63 (95%CI 0.58-0.68); highest income, 1.27 (1.14-1.42) | Vaccine initiation increased with maternal income |
| Feiring et al^8^ | Norway | Maternal income | Multivariable risk difference | Vaccination | Education, employment, country of origin, urbanity, maternal age at daughter’s birth, number of siblings, region, year of birth | Lowest quintile | Highest maternal quintile, 10.1% (9.0% - 11.3%) | The highest maternal income quintile was associated with higher vaccine initiation compared to the lowest quintile |
| Bjerke et al^3^ | Norway | Household income | Multivariable risk difference | Vaccination | Country background, year of birth, parental education, number of siblings, maternal age at daughter’s birth, region | Lowest quintile | Highest maternal quintile, 4.9% (4.3%-5.5%) | Compared to girls in quintile 1 (lowest) for household income, higher quintiles were more likely to initiate HPV vaccination |
| Wang et al^27^ | Sweden | Family income quintiles | Adjusted Hazard Ratio | Vaccination | County of residence, education, country of birth | Highest quintile | Lowest quintile, 0.87 (0.85-0.88) | Lower vaccine uptake among lower socioeconomic status compared to higher socioeconomic status. |
| Wemrell et al^28^ | Sweden | Parental income | Adjusted Odds Ratio | Non-vaccination | Parental education, parental country of birth, region, random effects | High income | Low income, 1.73 (95%CI: 1.69-1.78) | Non-vaccination was higher among girls with lower income parents compared to higher income parents. |
| Bedford et al^2^ | UK | Household income | Adjusted Odds Ratio | Vaccination | Parental ethnic background, religious faith, school exclusion, school type, age at interview | High income | Lowest income, 0.44 (95%CI 0.30-0.64) | Girls in the poorest households were less likely to have initiated vaccination compared to higher income households. |

**Supplementary Table 4. Strength of association between HPV vaccination and area-level socioeconomic status (SES) among included studies which identified an association between these variables**

| **Study** | **Country** | **Sociodemographic measure** | **Measure of association** | **Outcome** | **Adjusted for?** | **Reference group** | **Result** | **Summary** |
| --- | --- | --- | --- | --- | --- | --- | --- | --- |
| Mak et al^15^ | Australia | Relative socioeconomic disadvantage (RSED) based on school location | Odds ratio | Vaccination | Clustering by school | Least RSED | Most RSED, 0.7 (95%CI: 0.6-0.9)) | Lower vaccine uptake in most disadvantaged SES (68.8%) compared to least disadvantaged (75.1%) |
| Brotherton et al (2022)^5^ | Australia | ABS Socio-economic Index for Areas (SEIFA) quintiles | Chi square | Non-vaccination | N/A | Lowest SEIFA | 18.1% v 15.1%, p<0.0001 | Unvaccinated girls more likely to reside in areas of lowest SES quintile than vaccinated girls |
| Gertig et al^10^ | Australia | ABS Socio-economic Index for Areas (SEIFA) quintiles | Chi square | Non-vaccination | N/A | Lowest SEIFA | 18.4% v 15.5%, p<0.0001 | Unvaccinated girls more likely to have lower SES than vaccinated girls |
| Remes et al^20^ | Canada | Area deprivation index | Adjusted Odds Ratio | Vaccine refusal | Vaccination history, healthcare utilisation, medical history, income | Lowest quartile of deprivation | Highest area-level quartile, 0.82 (95%CI: 0.79-0.86) | Compared to those in the lowest quartile of area-level deprivation, those in the highest quartile (most deprived) were less likely to refuse vaccination |
| Poole et al^19^ | New Zealand | School decile (socioeconomic status) | Odds ratio | Vaccination | N/A | Decile 1 (poorest decile) | Decile 10, 5.72 (95%CI 3.36-9.71) | School-level SES was inversely associated with vaccination, with higher rates among the poorest decile (93%) compared to the wealthiest decile (66%). |
| Riesen et al^21^ | Switzerland | Socioeconomic position (neighbourhood level SEP) | Adjusted Odds Ratio | Vaccination | Nationality, urbanization, political opinion, religion, language, survey, school-based vaccination | Middle SEP | Lowest SEP, 1.18 (95%CI 1.00-1.38) | Vaccination uptake was higher in municipalities in the lower SES quartile, though there were not any differences between high and medium SES quartiles. |
| Roberts et al^22^ | UK | Index of multiple deprivation (IMD) 2010 | Odds ratio | Vaccination | N/A | Per 10-point increase in IMD | 0.89 (95%CI: 0.85-0.95) | Vaccine uptake was highest among girls living in the least deprived areas |
| Spencer et al^26^ | UK | Index of multiple deprivation (IMD) 2010 | Odds ratio | Vaccination | N/A | Least deprived | Most deprived, 1.09 (95%CI 1.00-1.17) | There were weak associations between vaccine initiation and area level deprivation (IMD), with higher uptake in the most deprived compared to least deprived |
| Fisher et al^9^ | UK | Index of multiple deprivation (IMD) 2010 | Adjusted Odds Ratio | Vaccination | Ethnicity, local authority, programme year, educational setting | Least deprived | Unadjusted OR, 3^rd^ quintile, 0.79 (0.66-0.93); unadjusted OR, 4^th^ quintile 0.68 (0.58-0.80) | The 3rd and 4th ^mo^st deprived quintiles were less likely to initiate vaccination compared to the least deprived in an unadjusted analysis, but this association did not remain in an adjusted analysis (p=0.48). |

**Supplementary Table 5. Strength of association between HPV vaccination and parental education among included studies which identified an association between these variables**

| **Study** | **Country** | **Sociodemographic measure** | **Measure of association** | **Outcome** | **Adjusted for?** | **Reference group** | **Result** | **Summary** |
| --- | --- | --- | --- | --- | --- | --- | --- | --- |
| Ogilvie et al^17^ | Canada | Parental education | Chi square | Vaccination | N/A | High school diploma | More than high school diploma, 63.3% v 72.9%, p<0.01 | Parents with more education (more than high school diploma/vocational training v high school diploma) were less likely to consent to their daughter being vaccinated |
| Hansen et al^12^ | Norway | Parental education | Adjusted Odds Ratio | Vaccination | Age, marital status, occupational status, maternal country of birth, children in household, maternal cervical screening, region, year of birth | Intermediate education | Primary school only, 1.76 (95%CI: 1.40-2.21); postgraduate, 1.40-2.21) | Vaccine initiation was higher among girls with less educated mothers and lower among highest education, compared to intermediate education. |
| Feiring et al^8^ | Norway | Parental education | Multivariable risk difference | Vaccination | Income, employment, country of origin, urbanity, maternal age at daughter's birth, number of siblings, region, year of birth | Compulsory education only | Highest education, -5.5% (95%CI -7.0% - -4.0%) | Highest maternal education was associated with a lower probability of being vaccinated compared to mother's with only compulsory education. |
| Bjerke et al^3^ | Norway | Parental education | Multivariable risk difference | Vaccination | Country background, year of birth, parental income, number of siblings, maternal age at daughter's birth, region | Compulsory education only | Undergraduate, -0.8% (-1.4% - -0.3%); graduate, -1.6% (-2.3% - -0.8%) | Girls with parents in higher education were less likely to initiate HPV vaccination compared to parents with less education. |
| Wang et al^27^ | Sweden | Parental education | Adjusted Hazard Ratio | Vaccination | County of residence, income, country of birth | High education | Low education, 0.92 (95%CI 0.91-0.94) | Vaccine uptake was lower among girls whose parents had low education compared to those with higher education |
| Wemrell et al^28^ | Sweden | Parental education | Adjusted Odds Ratio | Non-vaccination | Parental income, parental country of birth, region, random effects | High education | Low education, 1.73 (95%CI: 1.69-1.78) | Non vaccination was higher among girls whose parents had lower education compared to high education |

**Supplementary Table 6. Strength of association between HPV vaccination and religion among included studies which identified an association between these variables**

| **Study** | **Country** | **Sociodemographic measure** | **Measure of association** | **Outcome** | **Adjusted for?** | **Reference group** | **Result** | **Summary** |
| --- | --- | --- | --- | --- | --- | --- | --- | --- |
| Mak et al^15^ | Australia | School religion | Odds ratio | Vaccination | Clustering by school | Government schools | Catholic schools, 1.2 (95%CI 1.0-1.5) | Higher uptake among girls attending Catholic schools compared to government schools (77.3% v 73.1%). |
| Krawczyk et al^13^ | Canada | Parental religion | Chi square | Vaccination | N/A | Girls with non-Christian parents | Christian parents, 89.9% v 79.55%, p<0.01 | Girls with Christian parents were more likely to be vaccinated compared to non-Christian girls |
| Bowyer et al^4^ | UK | Religion | Adjusted Odds Ratio | Vaccination | Ethnicity | Christian girls | Unadjusted OR, No religion 1.77 (95%CI 1.16-2.70) | Girls with no religion were more likely to be fully vaccinated than Christian girls, but this association did not remain after adjustment. |
| Bedford et al^2^ | UK | Parental religion | Adjusted Odds Ratio | Vaccination | Parental ethnic background, income, school exclusion, school type, age at interview | No religious faith | Unadjusted OR, 0.76 (95%CI 0.59-0.97) | Girls whose parents reported any religious faith were less likely to be vaccinated, but this association did not remain after adjustment. |

**Supplementary Table 7. Strength of association between HPV vaccination and ethnicity/country of birth among included studies which identified an association between these variables**

| **Study** | **Country** | **Sociodemographic measure** | **Measure of association** | **Outcome** | **Adjusted for?** | **Reference group** | **Result** | **Summary** |
| --- | --- | --- | --- | --- | --- | --- | --- | --- |
| Krawczyk et al^13^ | Canada | Ethnicity | Chi square | Vaccination | N/A | Non-White girls | White girls, 89.9% v 74.6%, p<0.01 | White girls were more likely to be vaccinated compared to non-White girls |
| Gilbert et al^11^ | Canada | Parental country of birth | Adjusted Odds Ratio | Non-vaccination | Age, region, place of birth | Canadian-born parents | Parents born in Americas (not Canada), 1.69 (95%CI 1.03-2.77); Parents born in Europe, 2.66 (1.58-4.49) | Girls with parents born outside of Canada were more likely to not be vaccinated compared to girls with Canadian-born parents (no association for Africa/Asia/Oceania) |
| Poole et al^19^ | New Zealand | Ethnicity | Odds ratio | Vaccination | N/A | European | Pacific, 4.30 (95%CI 3.69-5.02) | Vaccination was highest among Pacific girls (88%) compared to Asian (79%), Maori (78%), and Europeans (63%) |
| Bjerke et al^3^ | Norway | Country of birth | Multivariable risk difference | Vaccination | Income, year of birth, parental education, number of siblings, maternal age at daughter's birth, region | Norwegian girls | Western Europe, -7.9% (95%CI: -9.1% - -6.1%); Central and Eastern Europe, -3.3% (-4.6% - -2.1%); Sub Saharan Africa, -3.4% (-5.0% - -1.8%); America and Oceania, -5.5% (-8.8% - -2.2%) | Girls from Western Europe, Central and Eastern Europe, Sub Saharan Africa, America and Oceania were less likely to initiate HPV vaccination compared to Norwegian girls. |
| Wang et al^27^ | Sweden | Country of birth | Adjusted Hazard Ratio | Vaccination | County of residence, education, income | Girls born in Sweden | Born outside of Sweden, 0.82 (95%CI 0.81-0.83) | Lower vaccine uptake was associated with girls born outside of Sweden compared to those born in Sweden |
| Wemrell et al^28^ | Sweden | Parental country of birth | Adjusted Odds Ratio | Non-vaccination | Parental education, parental income, region, random effects | Swedish-born parents | Immigrant background, 1.53 (95%CI 1.50-1.57) | Non vaccination was higher among girls who had parents with an immigrant background, compared to Swedish born parents |
| Bowyer et al^4^ | UK | Ethnicity | Adjusted Odds Ratio | Vaccination | Religion | White girls | Black, 0.41 (95%CI: 0.27-0.60); Other, 0.56 (0.38-0.82) | Girls from Black and Other ethnic groups were less likely to be fully vaccinated compared to White girls |
| Roberts et al^22^ | UK | Ethnicity | Odds ratio | Vaccination | N/A | White girls | Non-White, 0.67 (95% CI 0.49-0.92) | Non-White girls were less likely to be vaccinated than White girls, but there was no association by specific ethnic groups. |
| Spencer et al^26^ | UK | Area-level ethnicity | Odds ratio | Vaccination | N/A | White | Asian, 0.90 (95% CI 0.88-0.92); Black, 0.85 (0.77-0.94); Other, 0.65 (0.51-0.83) | Girls living in areas with high proportions of Asian, Black (OR 0.85 (0.77-0.94)), and Other ethnic groups were less likely to initiate vaccination. |
| Fisher et al^9^ | UK | Ethnicity | Adjusted Odds Ratio | Vaccination | IMD, local authority, programme year, educational setting | White girls | Asian/Asian British, 0.59 (95%CI 0.44-0.80); Black/Black British, 0.50 (0.32-0.79); Chinese/other, 0.48 (0.33-0.71) | Girls from Asian/British Asian, Black/Black British, and Chinese/Other ethnic groups were less likely to initiate HPV vaccination compared to White girls |
| Bedford et al^2^ | UK | Parental ethnicity | Adjusted Odds Ratio | Vaccination | IMD, income, school exclusion, school type, age at interview | White girls | Black African, 0.49 (95%CI 0.26-0.95); Any other, 0.43 (0.17-0.66)) | Young girls whose parents were from Black African or Any Other ethnic groups were less likely to be vaccinated compared to White girls. |

**References**

1 Munn Z, Moola S, Lisy K, Riitano D, Tufanaru C. Chapter 5: Systematic reviews of prevalence and incidence. In: Aromataris E, Munn Z, eds. JBI Manual for Evidence Synthesis. JBI, 2020.

2 Bedford H, Firman N, Waller J, Marlow L, Forster A, Dezateux C. Which young women are not being vaccinated against HPV? Cross-sectional analysis of a UK national cohort study. *Vaccine* 2021; **39**: 5934–9.

3 Bjerke RD, Laake I, Feiring B, Aamodt G, Trogstad L. Time trends in HPV vaccination according to country background: a nationwide register-based study among girls in Norway. *BMC Public Health* 2021; **21**: 854.

4 Bowyer H.L., Dodd R.H., Marlow L.A.V., Waller J. Association between human papillomavirus vaccine status and other cervical cancer risk factors. *Vaccine* 2014; **32**: 4310–6.

5 Brotherton J, Hendry A, Dey A, Hull BP, Beard F. HPV vaccination coverage: slightly improved two-dose schedule completion estimates and historical estimates lower on AIR than HPV Register. *Aust N Z J Public Health* 2022; **46**: 394–400.

6 Brotherton J.M.L., Malloy M., Budd A.C., Saville M., Drennan K.T., Gertig D.M. Effectiveness of less than three doses of quadrivalent human papillomavirus vaccine against cervical intraepithelial neoplasia when administered using a standard dose spacing schedule: Observational cohort of young women in Australia. *Papillomavirus Res* 2015; **1**: 59–73.

7 Carpiano RM, Polonijo AN, Gilbert N, Cantin L, Dube E. Socioeconomic status differences in parental immunization attitudes and child immunization in Canada: Findings from the 2013 Childhood National Immunization Coverage Survey (CNICS). *Prev Med* 2019; **123**: 278–87.

8 Feiring B, Laake I, Molden T, *et al.* Do parental education and income matter? A nationwide register-based study on HPV vaccine uptake in the school-based immunisation programme in Norway. *BMJ Open* 2015; **5**: e006422.

9 Fisher H, Audrey S, Mytton JA, Hickman M, Trotter C. Examining inequalities in the uptake of the school-based HPV vaccination programme in England: a retrospective cohort study. *J Public Health Oxf Engl* 2014; **36**: 36–45.

10 Gertig DM, Brotherton JML, Budd AC, Drennan K, Chappell G, Saville AM. Impact of a population-based HPV vaccination program on cervical abnormalities: a data linkage study. *BMC Med* 2013; **11**: 227.

11 Gilbert NL, Gilmour H, Dube E, Wilson SE, Laroche J. Estimates and determinants of HPV non-vaccination and vaccine refusal in girls 12 to 14 y of age in Canada: Results from the Childhood National Immunization Coverage Survey, 2013. *Hum Vaccines Immunother* 2016; **12**: 1484–90.

12 Hansen BT, Campbell S, Burger E, Nygard M. Correlates of HPV vaccine uptake in school-based routine vaccination of preadolescent girls in Norway: A register-based study of 90,000 girls and their parents. *Prev Med* 2015; **77**: 4–10.

13 Krawczyk A., Knauper B., Gilca V., *et al.* Parents’ decision-making about the human papillomavirus vaccine for their daughters: I. quantitative results. *Hum Vaccines Immunother* 2015; **11**: 322–9.

14 Lefevere E, Theeten H, Hens N, De Smet F, Top G, Van Damme P. From non school-based, co-payment to school-based, free Human Papillomavirus vaccination in Flanders (Belgium): a retrospective cohort study describing vaccination coverage, age-specific coverage and socio-economic inequalities. *Vaccine* 2015; **33**: 5188–95.

15 Mak DB, Bulsara MK, Wrate MJ, Carcione D, Chantry M, Efller PV. Factors determining vaccine uptake in Western Australian adolescents. *J Paediatr Child Health* 2013; **49**: 895–900.

16 Meghani H., Dubey V., Kadri O., Mathur A., Cameron J., Beckermann K. Factors contributing to uptake of the publicly-funded HPV vaccine in Toronto. *Int J Infect Dis* 2010; **14**: e452.

17 Ogilvie G, Anderson M, Marra F, *et al.* A population-based evaluation of a publicly funded, school-based HPV vaccine program in British Columbia, Canada: parental factors associated with HPV vaccine receipt. *PLoS Med* 2010; **7**: e1000270.

18 Pollock KG, Tait B, Tait J, *et al.* Evidence of decreased HPV vaccine acceptance in Polish communities within Scotland. *Vaccine* 2019; **37**: 690–2.

19 Poole T, Goodyear-Smith F, Petousis-Harris H, *et al.* Human papillomavirus vaccination in Auckland: reducing ethnic and socioeconomic inequities. *Vaccine* 2012; **31**: 84–8.

20 Remes O, Smith LM, Alvarado-Llano BE, Colley L, Levesque LE. Individual- and regional-level determinants of human papillomavirus (HPV) vaccine refusal: the Ontario Grade 8 HPV vaccine cohort study. *BMC Public Health* 2014; **14**: 1047.

21 Riesen M, Konstantinoudis G, Lang P, *et al.* Exploring variation in human papillomavirus vaccination uptake in Switzerland: a multilevel spatial analysis of a national vaccination coverage survey. *BMJ Open* 2018; **8**: e021006.

22 Roberts SA, Brabin L, Stretch R, *et al.* Human papillomavirus vaccination and social inequality: results from a prospective cohort study. *Epidemiol Infect* 2011; **139**: 400–5.

23 Shapiro GK, Tatar O, Knauper B, Griffin-Mathieu G, Rosberger Z. The impact of publicly funded immunization programs on human papillomavirus vaccination in boys and girls: An observational study. *Lancet Reg Health Am* 2022; **8**: 100128.

24 Sinka K, Kavanagh K, Gordon R, *et al.* Achieving high and equitable coverage of adolescent HPV vaccine in Scotland. *J Epidemiol COMMUNITY Health* 2014; **68**: 57–63.

25 Smith LM, Brassard P, Kwong JC, Deeks SL, Ellis AK, Levesque LE. Factors associated with initiation and completion of the quadrivalent human papillomavirus vaccine series in an Ontario cohort of grade 8 girls. *BMC Public Health* 2011; **11**: 645.

26 Spencer AM, Roberts SA, Brabin L, Patnick J, Verma A. Sociodemographic factors predicting mother’s cervical screening and daughter’s HPV vaccination uptake. *J Epidemiol Community Health* 2014; **68**: 571–7.

27 Wang J, Ploner A, Sparen P, *et al.* Mode of HPV vaccination delivery and equity in vaccine uptake: A nationwide cohort study. *Prev Med* 2019; **120**: 26–33.

28 Wemrell M, Vicente RP, Merlo J. Mapping sociodemographic and geographical differences in human papillomavirus non-vaccination among young girls in Sweden. *Scand J Public Health* 2022; : 14034948221075410.

29 Yeung A., Grulich A., Richters J., *et al.* Does hpv vaccine initiation influence sexual behaviour? findings from the second Australian study of health and relationships. *Sex Transm Infect* 2019; **95**: A180.
